# Groove strength is associated with cortical *β* suppression and tapping stability

**DOI:** 10.3389/fnhum.2026.1922766

**Published:** 2026-09-11

**Authors:** Kentaro Ono

**Affiliations:** Center for Brain, Mind, and KANSEI Sciences Research, Hiroshima University, Hiroshima, Japan

**Keywords:** beta-band activity, EEG, groove, oscillation, synchronized tapping

## Abstract

Groove—the pleasurable urge to move in response to rhythmic music—is known to engage motor planning networks in the brain, and *β* band oscillations over motor cortex correlate with groove strength during passive listening. Whether this groove-related *β* modulation extends to active sensorimotor synchronization, and whether it tracks behavioral synchronization performance, remains an open question. Here we tested whether groove ratings are associated with motor cortical *β* suppression and tapping stability during active synchronization and after auditory input ceases. Twenty-eight healthy adults tapped with the right index finger in time with drum rhythm patterns that varied in syncopation level (low, medium, high) while electroencephalography (EEG) was recorded. Following the tapping task, participants rated the groove strength of each pattern. *β* power over left central electrodes contralateral to the tapping hand was estimated separately for the synchronization phase, in which participants tapped to the rhythm without an explicit beat cue, and the continuation phase, in which tapping was maintained in silence. Groove ratings showed an inverted U-shaped relationship with syncopation level, peaking at intermediate syncopation. Tapping variability and *β* suppression over motor cortex showed the complementary pattern, both reaching their minimum at the same intermediate syncopation level. Rhythm patterns that were subsequently rated as more groove-inducing were associated with lower *β* power during both the synchronization and continuation phases, indicating that patterns rated as more groove-inducing were associated with stronger motor cortical suppression regardless of phase. These results indicate that groove is associated with greater motor cortical engagement during active sensorimotor synchronization, and that this engagement persists beyond the presence of auditory input, linking the subjective urge to move with more stable rhythmic motor control.

## 1 Introduction

Synchronizing movement to an auditory rhythm is a capacity so pervasive in humans that its neural mechanisms remain incompletely understood despite decades of research (Repp and Su, 2013). Sensorimotor synchronization—the coordination of rhythmic actions with external temporal cues—is typically studied via synchronized finger-tapping paradigms, in which participants tap in time with isochronous sequences or more complex rhythmic patterns (Repp, 2005). Synchronized tapping is not reactive: the motor system anticipates incoming beats rather than responding to them, as evidenced by the ubiquitous negative mean asynchrony in which taps precede beats by 20–100 ms (Kato and Konishi, 2006; Zendel et al., 2011; Ono et al., 2022; Rahimpour Jounghani et al., 2023; Pazdera and Trainor, 2025). This predictive character implies that sensorimotor synchronization depends on an internal model of temporal structure that continuously updates based on auditory feedback and prior expectations.

During active movement, *β* oscillation over sensorimotor cortex is a reliable marker of motor cortical engagement (Pfurtscheller and Lopes da Silva, 1999). Typically, *β* power decreases before and during a movement, representing the release of the motor system from inhibition (Pfurtscheller et al., 2003; Joundi et al., 2012). In tapping tasks, auditory pacing produces both stronger *β* suppression and more accurate tapping than visual pacing (Heijs et al., 2025). Active auditory-motor synchronization produces larger *β* modulations than passive listening, consistent with *β* serving as a neural signal of predictive temporal engagement during synchronized tapping (Engel and Fries, 2010; Schmidt-Kassow et al., 2023).

Groove—the pleasurable urge to move in synchrony with rhythm (Madison, 2006)—is known to engage the brain regions related to motor planning and execution (Matthews et al., 2020; Engel et al., 2022), and *β* oscillations over auditory and motor areas correlate with the sensation of groove during passive listening (Zalta et al., 2024). Unlike general rhythmic pleasure or perceived regularity, groove is specifically tied to auditory-motor coupling. Listeners describe it as a compelling pull toward synchronized bodily movement, and high-groove music reliably elicits more consistent and coordinated movement than low-groove music (Janata et al., 2012). However, groove introduces a dimension of variability into sensorimotor synchronization that has received little attention.

A defining feature of groove is its non-monotonic relationship with rhythmic complexity, particularly with respect to syncopation. Syncopation involves displacing accents onto metrically weak positions, often through the omission of notes on strong beats. Groove ratings peak at intermediate levels of syncopation and decline for both weakly and highly syncopated rhythms. This inverted U-shaped relationship between syncopation level and groove has been replicated across multiple stimulus sets and participant populations (Sioros et al., 2014; Witek et al., 2014, 2020; Senn et al., 2018; Matthews et al., 2019; Spiech et al., 2022; Pando-Naude et al., 2024), suggesting that groove is not simply a response to rhythmic predictability or surprise, but to a particular balance between them.

The characteristic interplay between groove and syncopation is often accounted for by the predictive coding perspective (Vuust et al., 2022; Matthews et al., 2023). In this framework, the brain continuously generates predictive models of the underlying beat and updates them based on the error between predicted and actual input. To minimize this error, the brain relies on active inference, recruiting the motor system to internally simulate and track the underlying beat. This heightened engagement of the motor network to resolve auditory uncertainty may manifest as the psychological urge to move, driving the stronger *β* suppression in sensorimotor cortex associated with groove.

Despite the specific link between groove and auditory-motor coupling and its known correlation with *β* oscillations, prior tapping research has often used metrically simple sequences that vary minimally in groove; groove research, conversely, has focused on passive listening or spontaneous movement rather than synchronized tapping. That *β* correlates with groove during passive listening (Zalta et al., 2024) raises the specific question of whether the same modulation operates during active tapping. Therefore, the aim of the present study is to clarify whether groove-related *β* modulation occurs during active sensorimotor synchronization in humans, and whether it tracks synchronization performance.

We hypothesized that groove ratings would be associated with cortical *β* suppression over contralateral motor cortex, consistent with greater motor cortical engagement, and that patterns rated as more groove-inducing would show stronger *β* suppression and better tapping synchronization accuracy and stability.

The present study tested these hypotheses. Participants tapped in time with rhythm patterns varying in syncopation level while EEG was recorded to assess *β* power (13–30 Hz) over left central electrodes (C1 and C3), reflecting motor cortex activity contralateral to the tapping hand, examined in relation to groove ratings, syncopation level, and tapping synchronization performance.

## 2 Materials and methods

### 2.1 Participants

Twenty-eight healthy undergraduate and graduate students [14 males and 14 females based on their self-report; age range 18–25 years, mean ± standard deviation (SD): 21.5 ± 2.1 years] were recruited through notices posted on the university campus. This study was designed and reported in accordance with the Sex and Gender Equity in Research (SAGER) guidelines (Heidari et al., 2016). Their musical experience varied (instrument playing years: range 0–14, mean ± SD: 3.3 ± 4.1), and none identified as professional musicians. Fifteen participants reported no musical training, and 13 reported some; among the latter, 10 played the piano and one each played the trombone, violin, or saxophone (Detailed demographic and musical background information is available in Supplementary Table 1).

The target sample size was determined using *a priori* power analysis using G* Power (version 3.1.9.7). To obtain sufficient sample size for an interaction between the factor of syncopation level (low, medium, high) and phase (Synchronization phase, Continuation phase; see Stimuli section) in a two-way repeated measures analysis of variance (ANOVA) with an expected medium effect size (*f* = 0.25), an alpha level of 0.05, and a desired power = 0.80, the analysis indicated a required sample size of 27 participants. Data were initially collected from 30 participants to exclude those who had poor data quality (e.g., excessive artifacts in EEG recordings). Based on these criteria, two participants were subsequently removed from the data analysis. Thus, the final sample subjected to data analysis consisted of 28 participants.

All participants were right-handed, assessed using the Japanese version of the FLANDERS Handedness Questionnaire (Okubo et al., 2014). Based on self-report, none had any motor, hearing, visual, or neurological impairments. Prior to participation, all participants provided written informed consent. The study protocol adhered to the Declaration of Helsinki and received approval from the local ethics committee of Hiroshima University (application No. E2024-0002).

### 2.2 Stimuli

The stimuli were selected from a pool of 50 rhythm patterns originally developed and validated by Witek et al. (2014). First, two patterns were reserved exclusively for the familiarization session prior to the main experiment. The remaining 48 patterns were then categorized into three syncopation levels (low, medium, high), with 16 patterns per level (Supplementary Table 2). This categorization was based on the syncopation index (SI) proposed by Witek et al. (2014), which considers both the metric position of each note, as defined by the monotonic model (Longuet-Higgins and Lee, 1984), and the polyphonic context of the drum kit. The SI calculation summed weights based on (1) the metric position of the syncopated note (stronger beats receive higher weight) and (2) the instrument producing the syncopation (bass or snare drum; see Supplementary text for details).

If all patterns were presented at a fixed tempo, participants could potentially achieve apparent synchronization by tapping at a constant, internally generated rate without attending to the rhythmic content. To prevent this, some trials were presented at slower (90 bpm) or faster (150 bpm) tempi; because participants could not predict the tempo of each trial, they were required to listen to each rhythm pattern and adjust their tapping rate accordingly. For each participant, the 16 patterns within each of the three syncopation levels were randomly assigned to one of three tempi: 10 patterns at 120 beats per minute (bpm), 3 patterns at 90 bpm, and 3 patterns at 150 bpm. Consequently, the main experiment comprised 48 trials (30 trials at 120 bpm, and 18 dummy trials at 90 or 150 bpm). Dummy trials were excluded from EEG and tapping data analyses.

Each pattern was reproduced in Finale software (version 26, MakeMusic, Louisville, USA) based on the original score transcriptions published in the supplementary material of Witek et al. (2014). Auditory stimuli were exported from Finale as WAV files (16-bit, 44.1 kHz) using Finale’s default MIDI drum kit (hi-hat, snare, and bass drum). All rhythm patterns were structured as two-bar phrases in 4/4 time. Syncopation was embedded within the bass and snare drum parts, while the hi-hat maintained a steady eighth-note pulse. All rhythm patterns are accessible at osf.io.^1^

### 2.3 Procedure

Participants were seated in a sound-attenuated room and listened to the rhythm patterns through insert earphones (ER4, Etymotic Research Inc., USA). Tapping was performed with the right index finger on a custom-made tapping device equipped with a small microphone, which detected tap sounds with a time resolution of approximately 1 ms.

**Figure 1:**
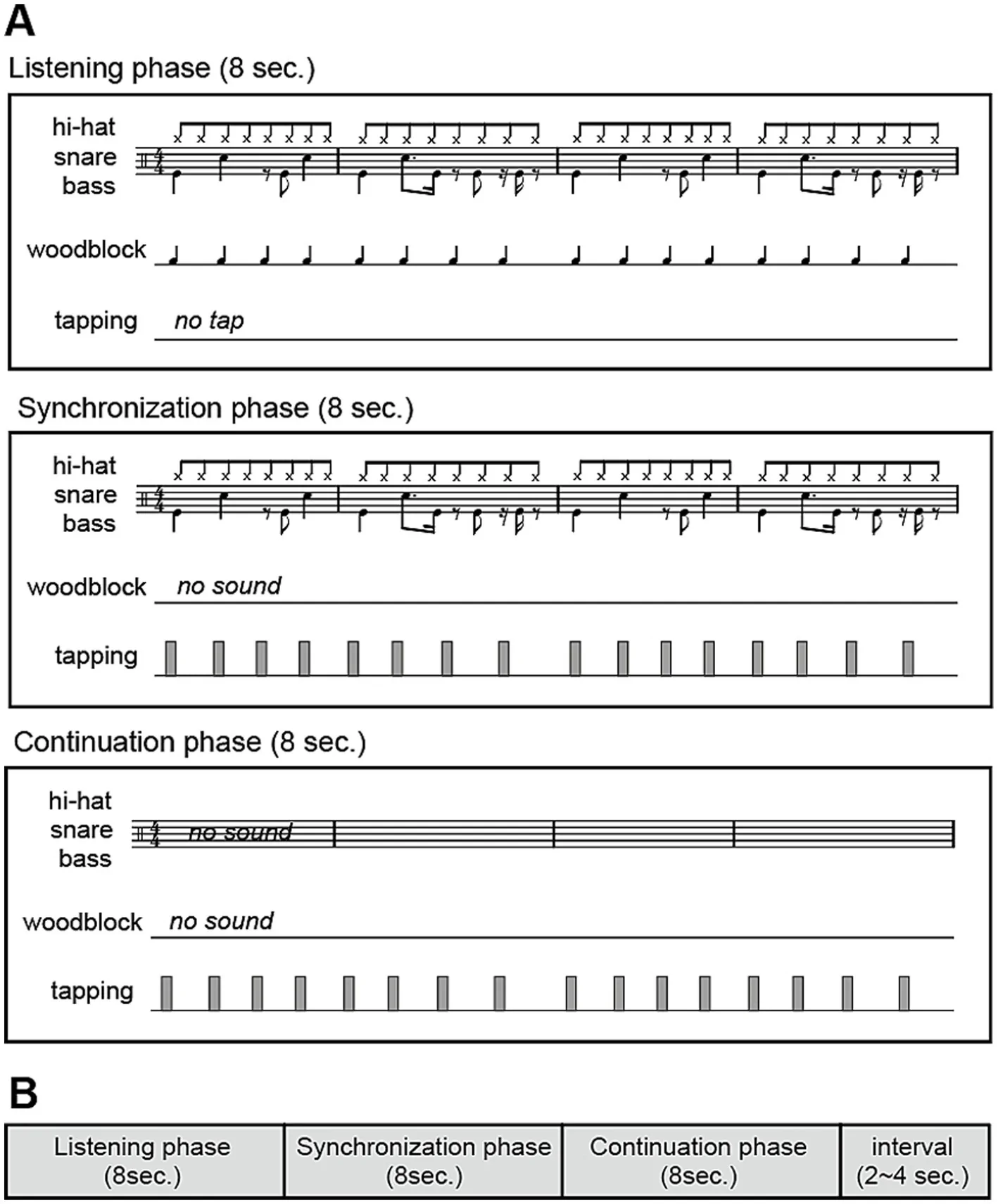
Experimental task structure **(A)** The three successive phases of each trial, shown with an example pattern (staves: hi-hat, snare, bass drum). Listening phase (8 s): the pattern was presented with woodblock sounds on every quarter-note beat, and participants listened without tapping. Synchronization phase (8 s): the woodblock stopped and participants tapped in synchrony with the rhythm. Continuation phase (8 s): all sounds ceased and participants maintained tapping in silence. **(B)** Trial timeline: the three phases followed by a 2–4 s silent inter-trial interval.

The experiment consisted of three consecutive sessions: a familiarization session, the main synchronized tapping session, and a rating session. The familiarization session used the two example rhythm patterns reserved from the original stimulus pool. The main synchronized tapping session used the remaining 48 rhythm patterns (including the varying tempi). Each trial in the familiarization and main tapping sessions was divided into three consecutive phases of equal duration (four bars each): a *Listening phase*, a *Synchronization phase*, and a *Continuation phase* (Figures 1A,B). For the 120 bpm trials, each phase lasted exactly 8 s (24 s per trial); dummy trials at 90 and 150 bpm differed in duration accordingly. Each trial began with the Listening phase, during which the two-bar rhythm pattern was presented twice (Figure 1A top). To explicitly guide beat perception, woodblock sounds were superimposed on all quarter-note beat positions throughout this phase. Participants were instructed to listen to the rhythm without tapping and internalize the underlying beat. The woodblock was intended to facilitate perception of the underlying beat prior to the Synchronization phase. In the subsequent Synchronization phase, the woodblock cue was discontinued, and participants were instructed to begin tapping in synchrony with the rhythm pattern alone (Figure 1A middle). Finally, in the Continuation phase, all auditory stimuli ceased, and participants were required to maintain their tapping at the same tempo established during the Synchronization phase (Figure 1A bottom). Trials were separated by a silent inter-trial interval that varied randomly between 2 and 4 s.

Finally, the rating session was conducted to obtain subjective evaluations for the 30 target rhythm patterns (120 bpm). Because patterns had been randomly assigned to tempi, the specific patterns at 120 bpm differed across participants. The rhythm patterns were presented once again in a random order, each lasting 16 s (corresponding to the combined duration of the Listening and Synchronization phases). Participants listened to each pattern without tapping. After the presentation of each pattern, participants rated the strength of the groove they experienced using a mouse-controlled visual analogue scale (VAS; 0 = no groove at all, 100 = very strong groove).

### 2.4 EEG measurement

Continuous EEG data were recorded using a BrainAmp DC amplifier (BrainProducts GmbH, Germany) and a 64-channel actiCAP electrode system (extended international 10–20 system) at a sampling rate of 1,000 Hz. The ground electrode was placed at Fpz, and data were referenced to an electrode placed on the nose tip during recording. Electrode impedances were kept below 10 kΩ. Electrooculography (EOG) was recorded for horizontal eye movements using electrodes placed at the outer canthi of both eyes; vertical EOG could not be placed as the electrode cap covered the supraorbital region.

### 2.5 EEG preprocessing and frequency analysis

Offline EEG data processing was performed using the EEGLAB toolbox (Delorme and Makeig, 2004). The raw data were down-sampled to 250 Hz, band-pass filtered between 1 Hz (high-pass) and 40 Hz (low-pass), and then re-referenced to the average of all scalp electrodes. Channels with poor signal quality were identified by visual inspection and interpolated using spherical spline interpolation. On average, 1.66 ± 2.0 [mean ± SD] channels per participant were interpolated. Then, independent component analysis (ICA), using the runica algorithm, was applied to identify and remove components related to eye blinks and muscle artifacts. Artifact-related components were identified using the MARA plug-in (Winkler et al., 2011, 2014) and removed if their artifact probability exceeded 0.5. On average, 22.4 ± 6.6 ICA components per participant were classified as artifacts and removed. Following component removal, the remaining data were visually inspected, and no epochs showed substantial residual artifacts. Because each rhythm pattern was presented only once per participant, no trials were rejected. Finally, data were segmented into epochs time-locked to the onset of the Listening phase, spanning from −2 s to 24 s (26 s trial). The period from −2 s to 0 s served as the baseline.

Power spectral density (PSD) was estimated separately for both Synchronization and Continuation phases using the Fieldtrip toolbox (Oostenveld et al., 2011). Data from each phase were extracted using 8-s time windows. PSD was computed using a multitaper FFT method with a Hanning taper at a frequency resolution of 1 Hz (1–45 Hz), and power values were log-transformed to decibels (10 × log10). Baseline correction was applied by subtracting the PSD computed over a 2-s silent window immediately preceding the Listening phase, yielding power values expressed as dB change from baseline, *β*-band power was defined as the mean power across 13–30 Hz at electrodes C1 and C3, averaged across the two channels, computed separately for each trial. These electrodes were selected *a priori* as left central sites contralateral to the right-hand tapping, where movement-related sensorimotor *β* modulation is classically maximal (Pfurtscheller and Lopes da Silva, 1999).

### 2.6 Tapping data analysis

Tapping data from the Synchronization and Continuation phases were subjected to analysis. During the Synchronization phase, although the explicit woodblock cue was removed, the underlying rhythm pattern was identical to that presented in the preceding Listening phase. Consequently, the temporal positions of the woodblock beats (four beats per bar) from the Listening phase were used as the implicit target beat onsets to calculate the time difference between the tap and the target beat. For the Continuation phase, where all auditory stimuli were completely absent, target beat onsets were defined by mathematically extrapolating the strict 500-ms (120 bpm) isochronous grid established in the preceding phases.

The mean and SD of time difference between the tap and the target beat, defined as mean and SD of asynchrony, were computed per trial per phase as measures of synchronization accuracy and stability, respectively.

### 2.7 Groove rating analysis

Groove ratings recorded on the VAS (0–100) were used without further transformation. For group-level comparisons, ratings were averaged across the 10 patterns within each syncopation level (low, medium, high) per participant. For correlation analyses, individual pattern-level ratings were used as a continuous variable.

### 2.8 Statistical analysis

All statistical analyses were performed in R (version 4.3.3) and RStudio (version 2024.12.1). Groove ratings were analyzed using one-way repeated measures ANOVA with syncopation level (low, medium, high) as the within-subjects factor, to verify that the syncopation manipulation produced the expected differences in perceived groove. Mean and SD of asynchrony and *β*-band power were each analyzed separately using two-way repeated measures ANOVAs with syncopation level (low, medium, high) and phase (Synchronization, Continuation) as within-subjects factors. Sphericity was assessed using Mauchly’s test; where the assumption was violated, degrees of freedom were corrected using the Greenhouse–Geisser method. Post-hoc pairwise comparisons were conducted using Shaffer’s modified sequentially rejective Bonferroni procedure (Shaffer, 1986). Effect sizes are reported as partial eta-squared (*eta*^2^) for ANOVA and Cohen’s *d* for post-hoc pairwise comparisons. The alpha level was set at 0.05 for all tests.

To examine whether groove ratings were associated with *β*-band power and tapping variability beyond syncopation, and whether any association differed between phases, we fitted linear mixed-effects models using the lmer4 and lmerTest packages in R. *β*-band power and the SD of asynchrony were each modelled as a function of syncopation index, groove rating (both z-scored), phase (Synchronization, Continuation), and the groove-by-phase interaction as fixed effects, with by-participant and by-rhythm pattern random intercepts. Multicollinearity was evaluated with variance inflation factors (VIF).

## 3 Results

### 3.1 Groove ratings

**Figure 2:**
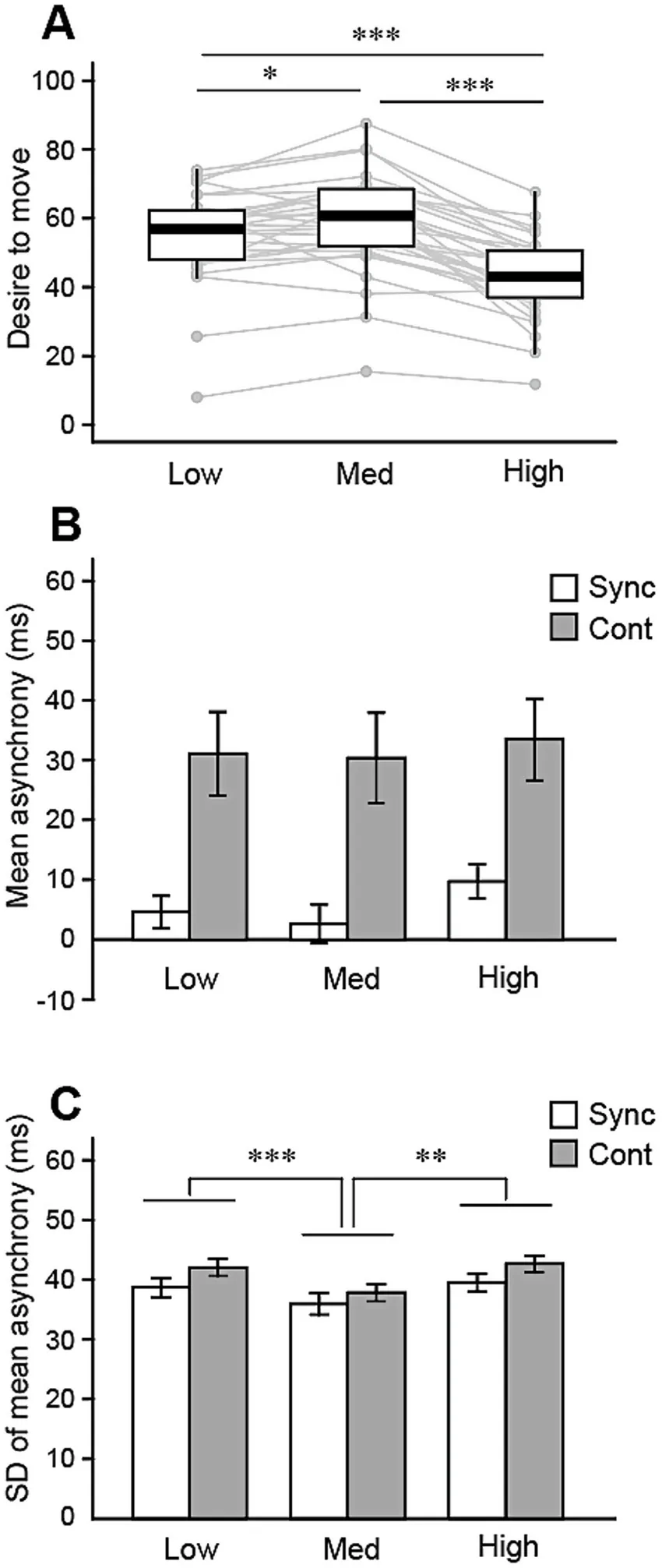
Groove ratings and tapping performance across syncopation levels. **(A)** Groove ratings (urge to move, 0–100) across three syncopation levels (Low, Medium, High). Boxes show interquartile range, horizontal lines within boxes indicate medians, and whiskers extend to 1.5 times the interquartile range. Individual participant data are presented as gray circle, connected by gray lines. **(B)** Mean asynchrony and **(C)** Standard deviation (SD) of asynchrony during the Synchronization (Sync, white bars) and Continuation phase (Cont, gray bars) across syncopation levels. Error bars indicate standard error of the mean (SEM). Asterisks denote significant post-hoc pairwise differences (*p < 0.05, **p < 0.01, ***p < 0.001).

Groove ratings showed an inverted-U shaped relationship with syncopation level (Figure 2A). A one-way repeated-measures ANOVA revealed a significant main effect of syncopation level (*F*_(2,54)_ = 43.54, *p* < 0.001, partial *eta*^2^ = 0.62). Post-hoc comparisons showed that the medium-syncopation pattern received higher ratings than both low-syncopation (*t*_(27)_ = 2.37, *p* = 0.025, *d* = 0.93) and high-syncopation patterns (*t*_(27)_ = 8.69, *p* < 0.001, *d* = 4.31). Additionally, low-syncopation patterns were rated higher than high-syncopation patterns (*t*_(27)_ = 6.16, *p* < 0.001, *d* = 3.41).

Acknowledging that a majority of participants reported no musical experience (15 of 28), we explored the potential impact by dividing participants into two groups: those who have no musical experience (N = 15) and those with some musical experience (*N* = 13). We performed separate two-way mixed ANOVAs on groove ratings with musical experience (no experience or some experience) as the between-subject factor and syncopation level as the within-subject factor. However, this analysis revealed neither significant main effect of musical experience (*F*_(1,26)_ = 0.07, *p* = 0.795, partial *eta*^2^ = 0.00) nor interaction (*F*_(2,52)_ = 0.48, *p* = 0.623, partial *eta*^2^ = 0.02).

### 3.2 Tapping asynchrony

Mean and SD of asynchrony were separately analyzed using a two-way repeated-measures ANOVA with syncopation level (low, medium, high) and phase (Synchronization, Continuation) as within-subjects factors. The ANOVA for mean asynchrony showed a significant main effect of phase (*F*_(1,27)_ = 16.79, *p* < 0.001, partial *eta*^2^ = 0.38) (Figure 2B). On the other hand, the ANOVA for SD of asynchrony showed a main effect of syncopation level (*F*_(2,54)_ = 7.93, *p* = 0.001, partial *eta*^2^ = 0.23). Post-hoc comparisons revealed that medium-syncopation pattern produced lower SD than both low-syncopation (*t*_(27)_ = 4.00, *p* = 0.001, *d* = 1.13) and high-syncopation patterns (*t*_(27)_ = 3.13, *p* = 0.004, *d* = 1.24) (Figure 2C).

To assess whether SD of asynchrony tracked groove beyond syncopation, we fitted a linear mixed-effects model with SI, groove ratings, phase, and their interaction as fixed effects and by-participant and by-pattern random intercepts. Groove rating was a significant negative predictor (*b* = −1.26, SE = 0.58, *t* = −2.16, *p* = 0.031), indicating lower tapping variability for patterns rated subsequently as more groove-inducing, beyond SI (*b* = −0.43, SE = 0.75, *t* = −0.58, *p* = 0.57). Tapping variability was higher in the Continuation than the Synchronization phase (*b* = 5.00, SE = 0.74, *t* = 6.74, p < 0.001), and the groove-by-phase interaction was not significant (*b* = 0.39, SE = 0.74, *t* = 0.53, *p* = 0.60) (random effects: participant variance = 3.38, pattern variance = 12.9, residual = 231.3).

To verify that the tapping-stability effect did not arise from cumulative tempo or phase drift during continuation, we additionally analyzed the SD of inter-tap interval (ITI), which is insensitive to such drift, using the same two-way ANOVA. The SD of ITI showed a significant main effect of syncopation level (*F*_(2,54)_ = 3.86, *p* = 0.027, partial *eta*^2^ = 0.13), with the lowest variability at medium syncopation (medium < low, *t* = 2.34, *p* = 0.030; medium < high, *t* = 2.77, *p* = 0.030), paralleling the SD of asynchrony.

### 3.3 *β*-band power in EEG

**Figure 3:**
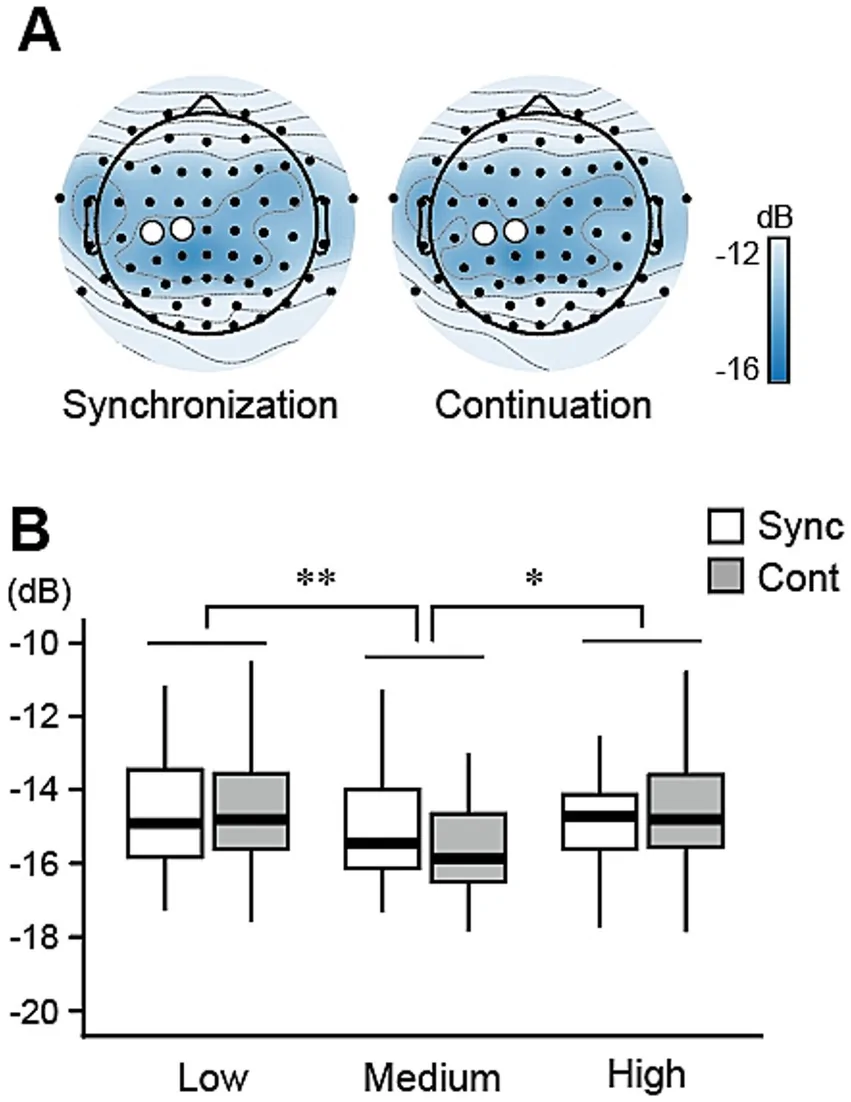
Motor cortical *β*-band power across syncopation levels and phases **(A)** Topographic maps of *β*-band power (13–30 Hz, dB change from baseline) averaged across participants, syncopation levels, and trials for the synchronization and continuation phases. White circles indicate the electrode used for analysis (C1 and C3) **(B)** *β*-band power at C1/C3 during the synchronization (sync, white boxes) and continuation (cont, gray boxes) phases across syncopation level. Boxes show interquartile range, horizontal lines within boxes indicate medians, and whiskers extend to 1.5 times the interquartile range. Asterisks denote significant *post-hoc* pairwise differences (*p < 0.05, **p < 0.01).

To examine whether motor cortical activity during tapping varied with syncopation level and phase, *β*-band power (13–30 Hz) at C1 and C3, expressed as dB change from baseline, was analyzed using the same two-way repeated-measures ANOVA. Topographic maps revealed that *β* suppression extended bilaterally across central and parietal regions in all three phases, with C1 and C3 positioned within this suppression area (Figure 3A). The ANOVA revealed the main effect of syncopation level (*F*_(2,54)_ = 4.54, *p* = 0.015, partial *eta*^2^ = 0.14). Post-hoc comparisons showed that the medium-syncopation pattern produced lower *β* power than both low-syncopation (*t*_(27)_ = 3.39, *p* = 0.006, *d* = 0.32) and high-syncopation patterns (*t*_(27)_ = 2.21, *p* = 0.036, *d* = 0.25), indicating greater motor cortical suppression at intermediate syncopation levels (Figure 3B).

The same mixed-effects model applied to *β* power showed a parallel pattern with SD of asynchrony. Groove rating was again a significant negative predictor of *β* power (*b* = −0.17, *SE* = 0.09, *t* = −2.00, *p* = 0.045), indicating stronger suppression for patterns subsequently rated as more groove-inducing, whereas SI did not explain additional variance (*b* = −0.07, *SE* = 0.08, *t* = −0.81, *p* = 0.43). Neither the main effect of phase (*b* = −0.12, *SE* = 0.11, *t* = −1.09, *p* = 0.28) nor the groove-by-phase interaction (*b* = −0.10, *SE* = 0.11, *t* = −0.96, *p* = 0.34) was significant, indicating the groove-*β* association did not differ between the Synchronization and Continuation phases (random effects: participant variance = 3.90, pattern variance = 0.12, residual = 4.91).

Because the topographic maps showed *β* suppression distributed broadly across bilateral central and parietal regions (Figure 3A), we examined whether the effect at C1/C3 was specific to the contralateral hemisphere by comparing *β* power at these electrodes with that at the ipsilateral homologues C2 and C4. A three-way ANOVA with the factors of hemisphere (left, right), phase (Synchronization, Continuation), and syncopation level (low, medium, high) showed no main effect of hemisphere (*F* (1,27) = 2.65, *p* = 0.11, partial *eta*^2^ = 0.09), consistent with broad bilateral suppression, but a significant hemisphere × syncopation interaction (*F*_(2, 54)_ = 3.21, *p* = 0.048, partial *eta*^2^ = 0.11). Decomposing this interaction, simple main effect analysis indicated that the effect of syncopation level was significant over the contralateral electrodes (C1/C3: *F*_(2,54)_ = 8.34, *p* < 0.001, partial *eta*^2^ = 0.24), but not over the ipsilateral (C2/C4: *F*_(2,54)_ = 1.89, *p* = 0.16, partial *eta*^2^ = 0.07). Further multiple comparison revealed greatest suppression at medium syncopation level at the contralateral electrodes (medium < low: *t* (27) = 4.59, *p* < 0.001; medium < high: *t* (27) = 3.11, *p* = 0.004). Correspondingly, contralateral suppression exceeded ipsilateral suppression at medium syncopation level (*F*_(1,27)_ = 5.05, *p* = 0.033, partial *eta*^2^ = 0.16), with no hemispheric difference at low or high syncopation.

## 4 Discussion

We measured EEG while participants tapped in synchrony with drum rhythm patterns that varied in syncopation level, and obtained groove ratings for each pattern. Groove ratings peaked at intermediate syncopation, and at the same intermediate level tapping variability was lowest and motor cortical *β* power was most strongly suppressed. Across rhythm patterns, higher groove ratings were associated with stronger *β* suppression and greater tapping stability, beyond the contribution of syncopation level, during both the synchronization and continuation phases. The inverted-U shaped relationship between syncopation and groove replicates previous studies (Sioros et al., 2014; Witek et al., 2014; Senn et al., 2018; Matthews et al., 2019; Spiech et al., 2022; Pando-Naude et al., 2024), and the selective reduction of tapping variability agrees with Janata et al. (2012) and the recent report of Zampini et al. (2025). By showing that the groove-*β* relationship established for passive listening (Zalta et al., 2024) also holds during active tapping and persists after auditory input ceases, our results extend this literature to active sensorimotor synchronization.

### 4.1 Non-monotonic modulations of groove ratings, tapping, and beta-band power

Groove ratings showed the inverted U-shaped relationship with syncopation level reported in Witek et al. (2014) and replicated across multiple subsequent studies (Sioros et al., 2014; Senn et al., 2018; Matthews et al., 2019; Witek et al., 2020; Spiech et al., 2022; Pando-Naude et al., 2024). Recent evidence further links this relationship to reward and dopaminergic function. Whereas healthy listeners show peak groove at moderate complexity, individuals with substance use disorders experience heightened groove at high complextity (Stupacher et al., 2013). Because the stimuli were drawn directly from the Witek et al. (2014) validated set, this result was anticipated; the large effect size (partial *eta*^2^ = 0.62) confirms that the three syncopation levels produced clearly differentiated groove experiences.

Tapping performance also showed the same non-monotonic pattern. Specifically, tapping stability, indexed by the SD of asynchrony, showed a significant effect of syncopation level. This behavioral alignment with groove ratings corroborates previous findings that high-groove music facilitates more consistent and coordinated bodily movement (Janata et al., 2012). Crucially, our results closely parallel recent evidence by Zampini et al. (2025) (preprint), who demonstrated that higher groove specifically reduces tapping variability rather than altering mean asynchrony. This selective enhancement of tapping stability can be accounted for by conceptualizing rhythm synchronization as a probabilistic inference process as formalized in the learning progress hypothesis (Matthews et al., 2023). Within the predictive coding framework, the brain models upcoming beat onsets as probability distributions, where the mean represents the expected timing and the variance reflects predictive uncertainty. Mean asynchrony indexes systematic biases in the mean of this distribution, arising from relatively automatic processes such as sensory latency compensation or phase correction, rendering it largely insensitive to variations in groove intensity. Conversely, the SD of asynchrony reflects the width of the predictive distribution, serving as a behavioral proxy for the precision of the internal model. Matthews et al. (2023) argue that intermediate syncopation maximizes “reducible prediction errors,” signaling an optimal potential for learning progress. To resolve this manageable temporal uncertainty and update the metrical model, the brain dynamically recruits the motor system via active inference. This recruitment manifests as the pleasurable urge to move. The resulting motor engagement stabilizes the internalized beat representation, narrowing the predictive distribution and thereby minimizing beat-to-beat timing variability. Because the baseline accuracy of beat detection (mean asynchrony) operates independently of this active, error-minimizing update process, it remains constant across syncopation levels.

In the present data, this behavioral optimization of predictive certainty was paralleled by neurophysiological dynamics within the motor cortex. Consistent with the tapping stability data, *β*-band power over the contralateral motor cortex (C1 and C3 electrodes) was lowest at the medium syncopation level. Specifically, medium-syncopation rhythms induced significantly lower *β* power, indicating the strongest cortical suppression, than either low- or high-syncopation rhythms. Because *β* suppression over the sensorimotor cortex is a marker of motor cortical engagement and the release from functional inhibition (Pfurtscheller and Lopes da Silva, 1999; Engel and Fries, 2010; Joundi et al., 2012), this maximal suppression is consistent with greater motor engagement at intermediate syncopation. However, *β* suppression is not specific to groove. It can also reflect motor execution, attention, prediction, effort, and general task engagement, and should not be interpreted as a direct or exclusive marker of groove-driven engagement. Although overall *β* suppression was broadly bilateral, its modulation by syncopation was specific to the contralateral hemisphere. Thus, while the overall response was bilateral, the syncopation-related component was consistent with engagement of contralateral sensorimotor cortex.

The functional significance of this motor engagement can be understood through the framework of predictive timing. As Arnal (2012) proposes, the motor system actively simulates future events to anticipate their occurrence, generating efferent signals (top-down predictions) that propagate to sensory areas. In this context, *β*-band oscillations mediate these descending predictive signals to constrain auditory processing (Arnal, 2012). As a result, when intermediate rhythmic complexity presents an optimal challenge, the motor network is most heavily engaged to internally simulate the beat. This active simulation may be reflected in the maximal *β* suppression, reflecting the robust generation of efferent signals necessary to resolve temporal uncertainty and update the metrical model. While recent studies established that sensorimotor *β* oscillations associate with groove during passive listening (Zalta et al., 2024; Zampini et al., 2025), our results extend this evidence to active synchronization. By demonstrating that maximal *β* suppression coincides exactly with the lowest tapping variability, our data are consistent with the view that the psychological urge to move may be related to a state of increased motor preparation that generates precise top-down predictions, fine-tuning ongoing actions. Consistent with the view that the motor component of groove is separable from its hedonic component, the urge to move is preserved in people with musical anhedonia despite their reduced musical pleasure (Romkey et al., 2025), supporting our focus on the motor correlates of groove.

### 4.2 Maintenance of groove in the continuation phase

A notable finding of the present study is that the association between rated groove and motor cortical engagement extended beyond the presence of auditory stimuli. During the Continuation phase, where participants maintained the tapping tempo in silence, the association between passively rated groove and *β* suppression remained significant. This persistence has theoretical implications regarding the nature of groove. It suggests that the urge to move may not be merely a transient, stimulus-driven response to incoming rhythmic complexity. Rather, it may reflect the quality and stability of an internalized temporal representation (Zampini et al., 2025).

Previous studies have demonstrated that primary sensorimotor cortex activation and motor *β* oscillatory dynamics exhibit equivalent levels of engagement during both externally paced synchronization and internally driven continuation (Rao et al., 1997; Liu et al., 2024). Rao et al. (1997) suggested that the primary sensorimotor cortex and cerebellum form a core circuit responsible for the fundamental execution of rhythmic tapping, functioning independently of whether the pacing cue is overt or internally maintained. Relatedly, Liu et al. (2024) indicated that *β* oscillations within this network consistently mediate both active motor execution and temporal prediction across both externally paced and internally driven tapping tasks. Building upon this evidence and the probabilistic and active inference frameworks, the sustained *β* suppression observed in the Continuation phase indicates that an optimized predictive model remains active in the absence of external input.

As proposed by Arnal (2012), in the absence of bottom-up sensory input, the motor system must rely primarily on endogenous efferent signals to maintain predictive timing. The continued association between high groove and strong *β* suppression in silence is consistent with the possibility that rhythms which more strongly engage the motor system support more precise internal timekeeping that persists after the external stimulus ceases, contributing to more stable tapping during continuation. Consistent with this, greater stability of neural oscillations has recently been shown to accompany more accurate auditory-motor synchronization (Scheurich et al., 2025), suggesting that the precision of internally maintained timing is reflected in the stability of ongoing neural dynamics. This interpretation remains tentative, as any internalized temporal representation was not directly assessed in the present study.

### 4.3 Limitations

Several limitations of the present study should be acknowledged. First, our participant pool was restricted to healthy young adults who were non-professional musicians. While groove perception seems less experience-dependent (Stupacher et al., 2013; Senn et al., 2018; Swarbrick et al., 2018), neural mechanisms could differ in more experienced musicians or across cultural groups with varying exposure to syncopated music (Witek et al., 2020). Future work, including musicians with formal training or participants from non-Western musical traditions could determine whether the groove-*β* relationship generalizes beyond the population studied here.

Second, the use of FFT to estimate *β* power over the full 8-s analysis window precluded any characterization of within-phase temporal dynamics. The resulting measure reflects average *β* power across each phase and cannot distinguish sustained suppression throughout the phase from phasic suppression time-locked to individual taps. Although tap-locked time-frequency analysis was considered, it was not implemented due to a technical constraint imposed by approximately 2 Hz tapping rate. At the lower end of the *β* band (13 Hz), wavelet kernels with adequate spectral resolution require a temporal window of approximately 500 ms or more (e.g., seven cycles at 13 Hz ≈ 538 ms), which approaches or exceeds the inter-tap interval. This overlap makes it difficult to isolate the *β* dynamics associated with a single tap from those of adjacent taps without substantial contamination. Phase-averaged FFT was therefore adopted as a more conservative approach. Whether the groove effect on *β* operates tonically across the entire phase or is concentrated around individual tap onsets remains an open question, and our interpretation of this effect as a predictive motor process should be regarded as provisional pending tap-locked analyses.

Third, our subjective and neurophysiological measures were temporally separated. To prevent dual-task interference and preserve the continuity of synchronized tapping, groove ratings were collected in a separate, passive-listening session after the main experiment. Consequently, our findings are most accurately described as a relationship in which rhythms later rated as more groove-inducing were associated with stronger *β* suppression during tapping. Because active movement itself is known to substantially amplify the sensation of groove, the magnitude or dynamic range of the groove experienced during tapping may have differed from that reported during passive listening.

A further limitation concerns sex difference. With 14 male and 14 female participants, the study was not powered to test for sex differences. Whether the observed associations differ between males and females remains open for adequately powered studies.

## 5 Conclusion

The present study showed, during active synchronization, rhythm patterns rated as more groove-inducing were accompanied by stronger cortical *β* suppression and steadier tapping, beyond the effect of syncopation. This coupling persisted after the rhythm stopped. Groove thus appears linked to the motor system not only during listening but also during active timekeeping.

## Data Availability

The raw data supporting the conclusions of this article will be made available by the authors, without undue reservation.

## Footnotes

1
https://osf.io/d4nj7

## Supplementary material

SUPPLEMENTARY TABLE 1 Participant demographic and musical background information. Participant ID, sex, age, years of musical experience, and primary musical instrument are listed for each of the 28 participants.

SUPPLEMENTARY TABLE 2 Rhythm patterns used in the experiment. All 48 rhythm patterns are listed in ascending order of syncopation index (SI). Each row shows the pattern number (No), SI value, and the corresponding two-bar rhythm sequence in musical notation. The top staff shows the hi-hat part; the bottom staves show the snare drum and bass drum parts. Patterns were categorized into three syncopation levels (low, medium, high) with 16 patterns per level based on SI values, as described in the Stimuli section.
