# Supplementary material for: Groove strength is associated with cortical β suppression and tapping stability
Source: Front Hum Neurosci. 2026 Sep 11;20:1922766. doi: 10.3389/fnhum.2026.1922766 (PMC13612199; doi:10.3389/fnhum.2026.1922766)
Supplement: Supplementary file 1 [file Data_sheet_1.pdf]

Supplementary Table 1. Participant's demographic information

Participant ID	Sex	Age	Years of musical experience	Musical instrument
S001	Male	23	0	*none*
S002	Male	20	0	*none*
S003	Male	25	0	*none*
S004	Male	24	0	*none*
S005	Male	24	6	Trombone
S006	Male	21	3	Violin
S007	Female	22	0	*none*
S008	Female	24	6	Sax
S009	Male	23	3	Piano
S010	Male	22	0	*none*
S011	Male	20	0	*none*
S012	Female	19	14	Piano
S013	Male	24	0	*none*
S014	Male	22	0	*none*
S015	Male	21	0	*none*
S016	Female	19	0	*none*
S017	Female	20	0	*none*
S018	Female	18	6	Piano
S019	Female	20	8	Piano
S020	Female	23	9	Piano
S021	Female	20	0	*none*
S022	Female	21	6	Piano
S023	Male	21	0	*none*
S024	Female	23	10	Piano
S025	Female	24	10	Piano
S026	Female	18	6	Piano
S027	Female	24	4	Piano
S028	Male	18	0	*none*
