# Supplementary material for: Groove strength is associated with cortical β suppression and tapping stability
Source: Front Hum Neurosci. 2026 Sep 11;20:1922766. doi: 10.3389/fnhum.2026.1922766 (PMC13612199; doi:10.3389/fnhum.2026.1922766)
Supplement: Supplementary file 2 [file Data_sheet_2.pdf]

No.	SI	Rhythm sequence
1	0	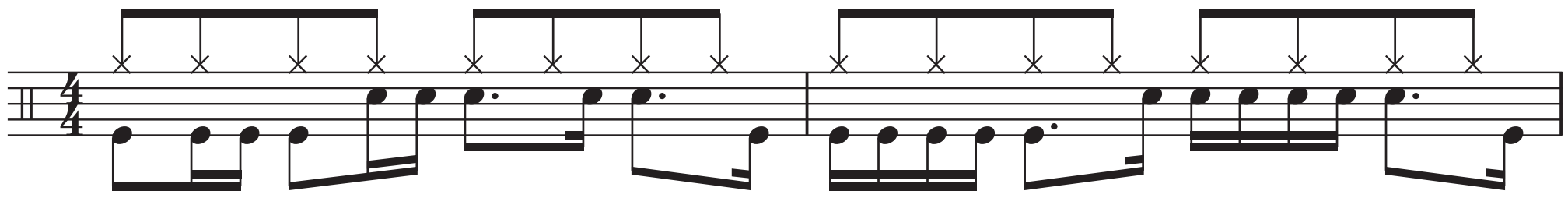
2	0	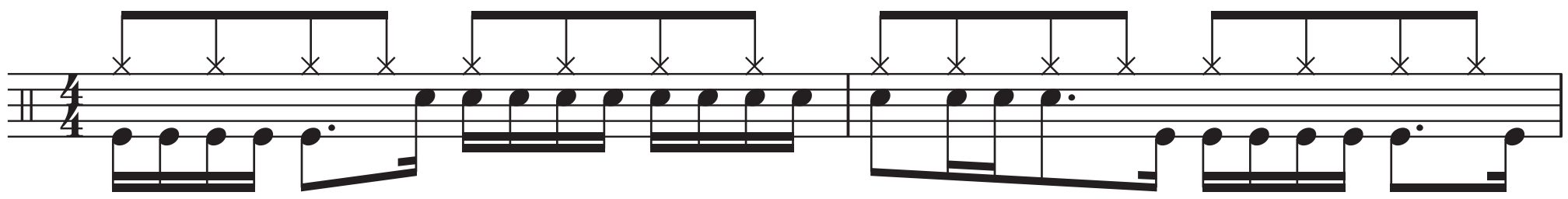
3	7	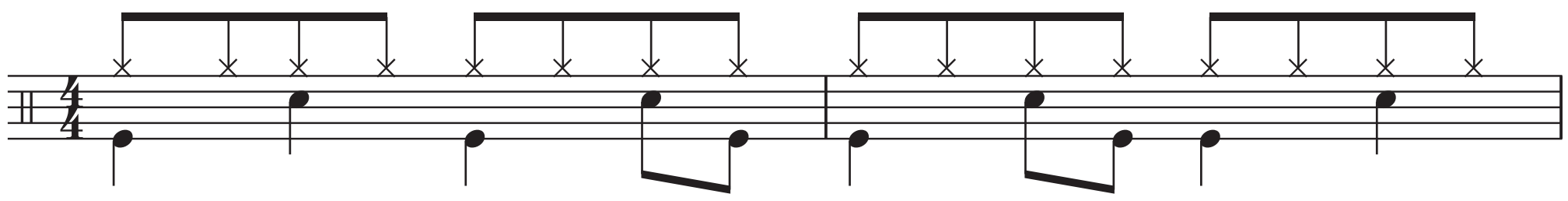
4	8	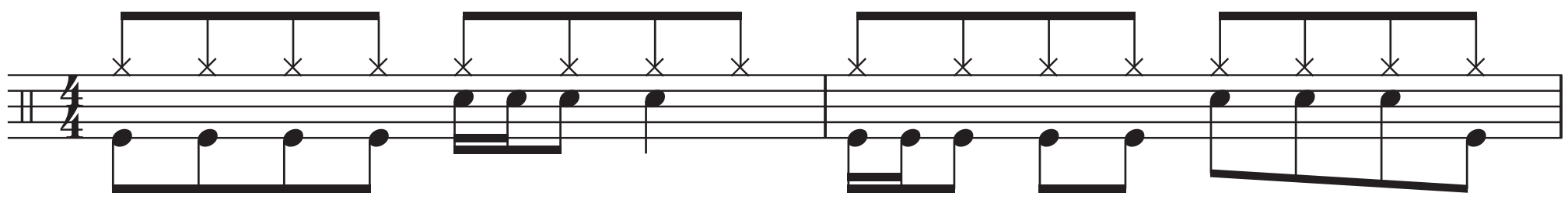
5	9	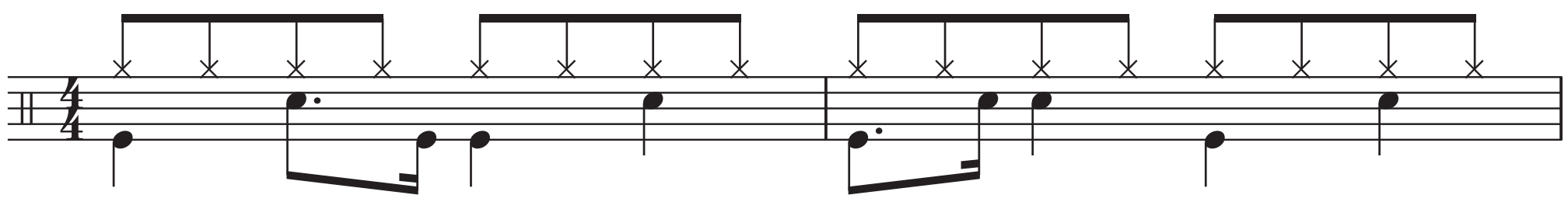
6	9	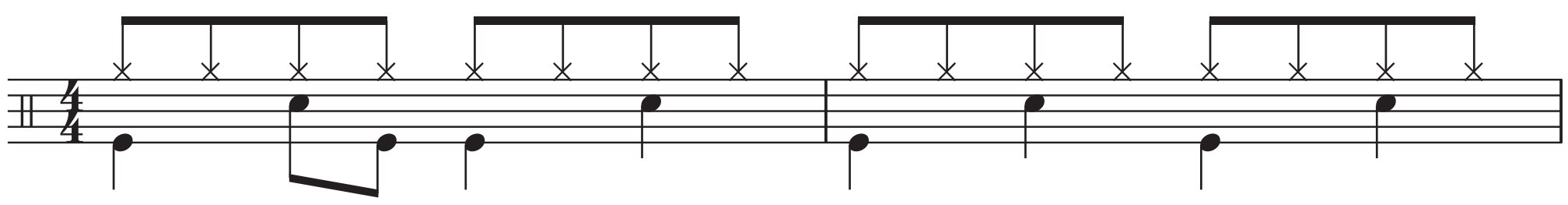
7	12	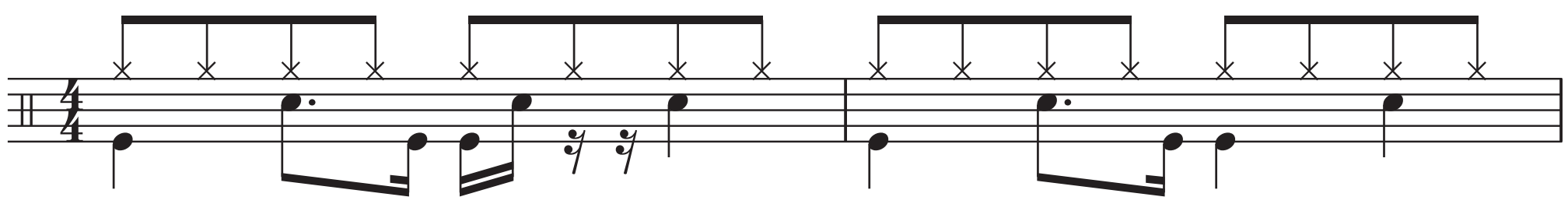
8	13	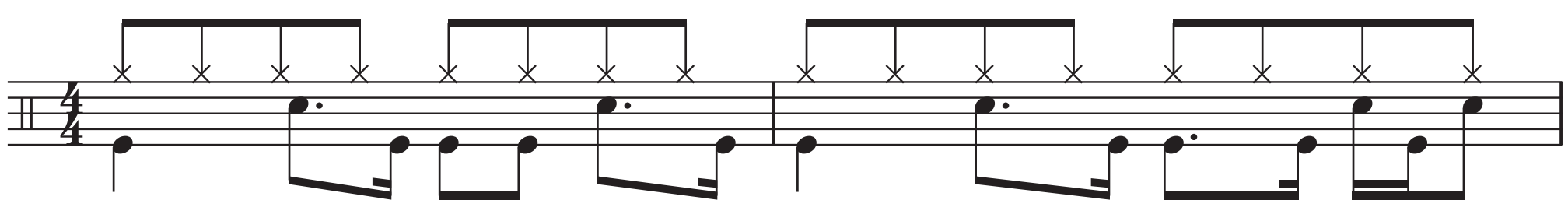
9	14	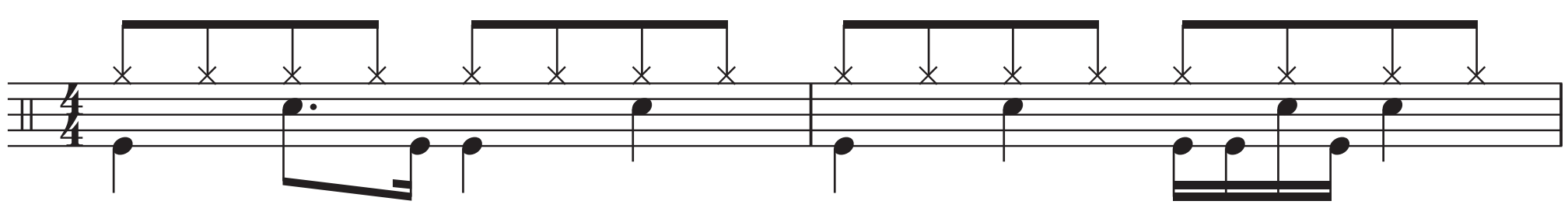
10	17	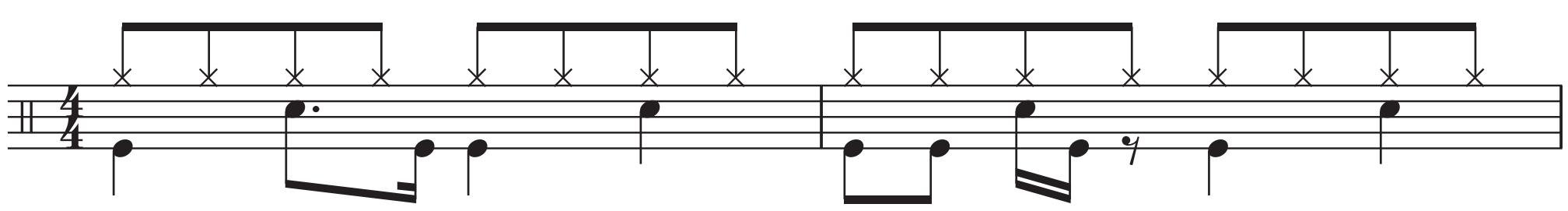
11	17	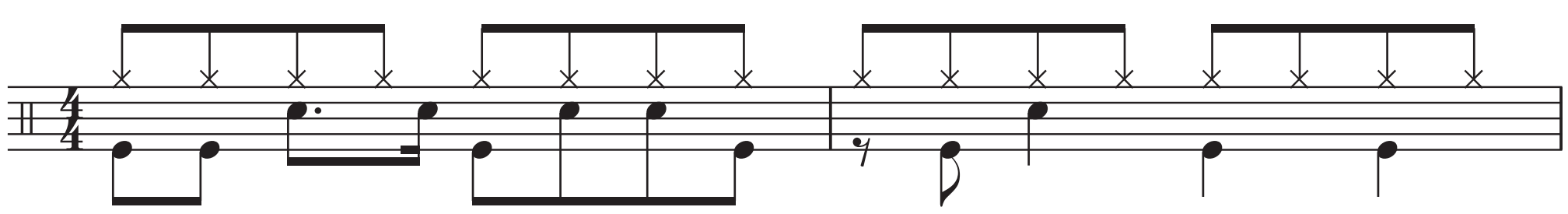
12	19	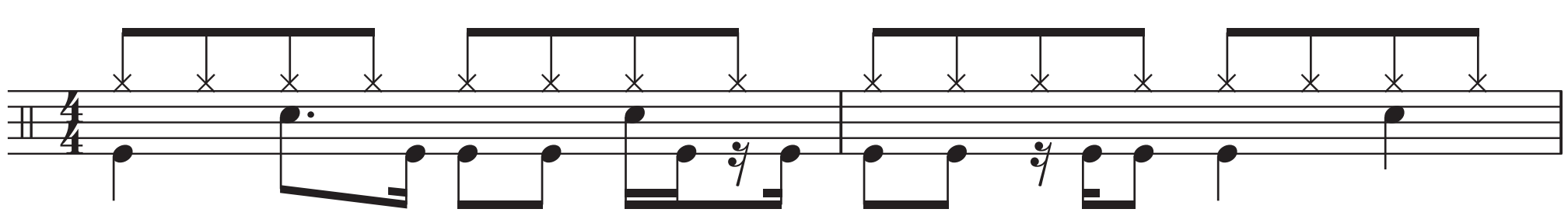

No.	SI	Rhythm sequence
13	21	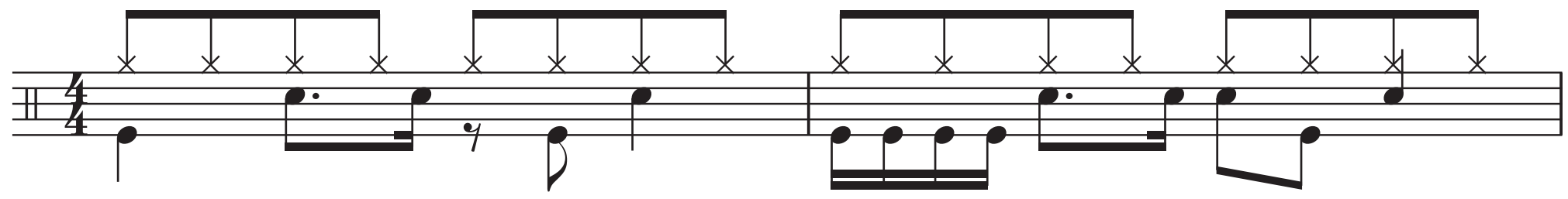
14	21	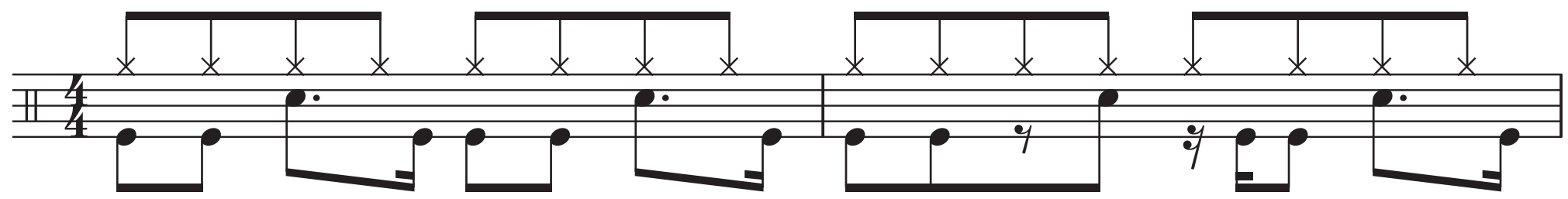
15	22	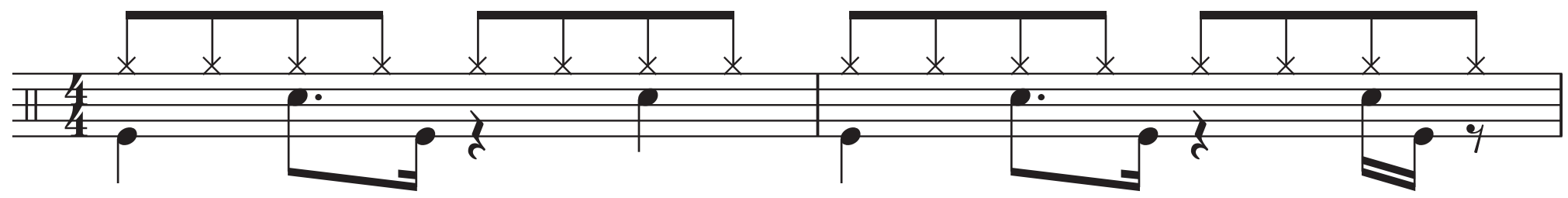
16	23	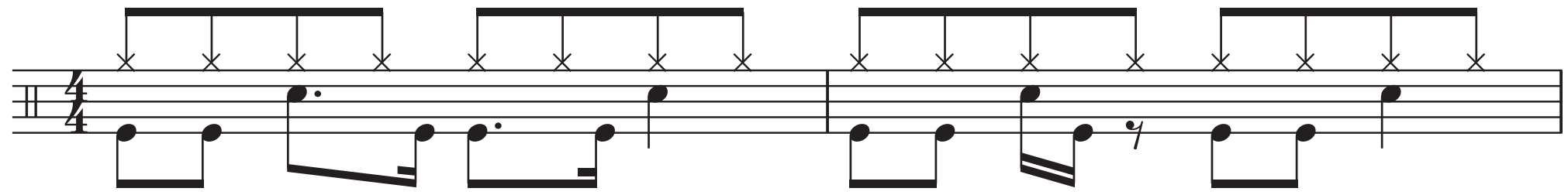
17	24	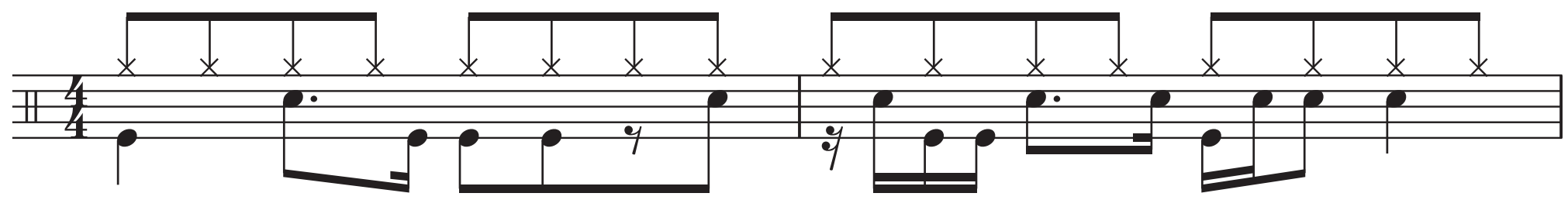
18	24	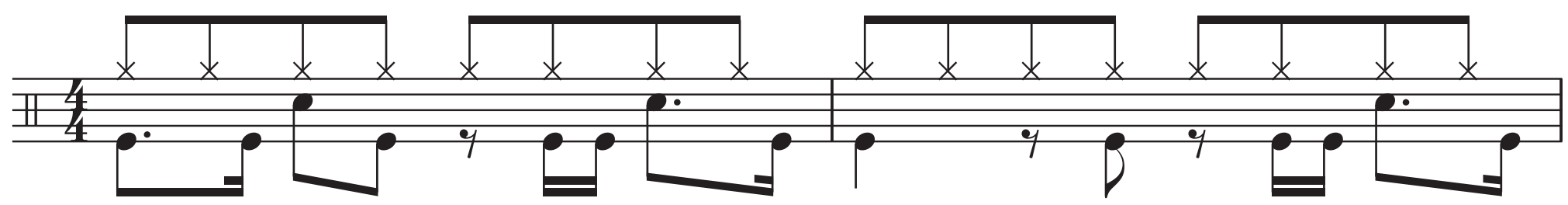
19	25	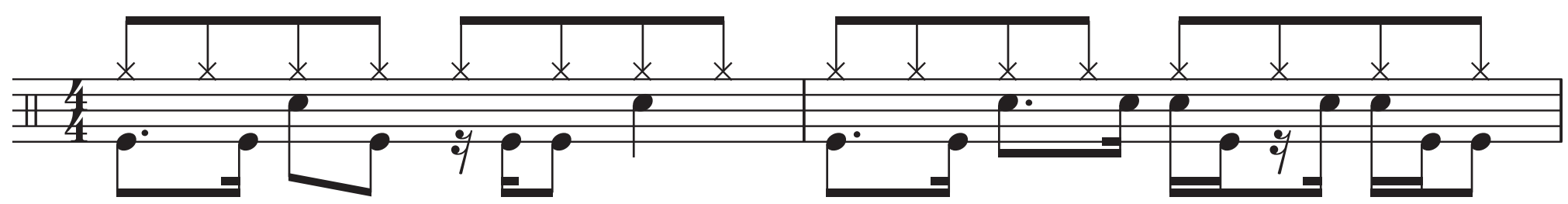
20	26	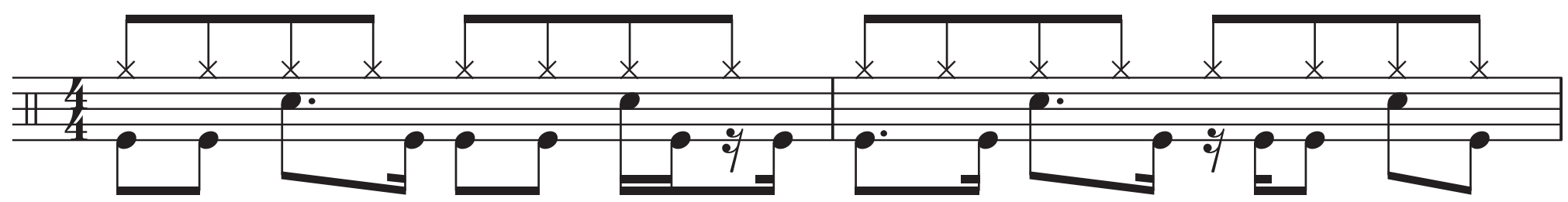
21	27	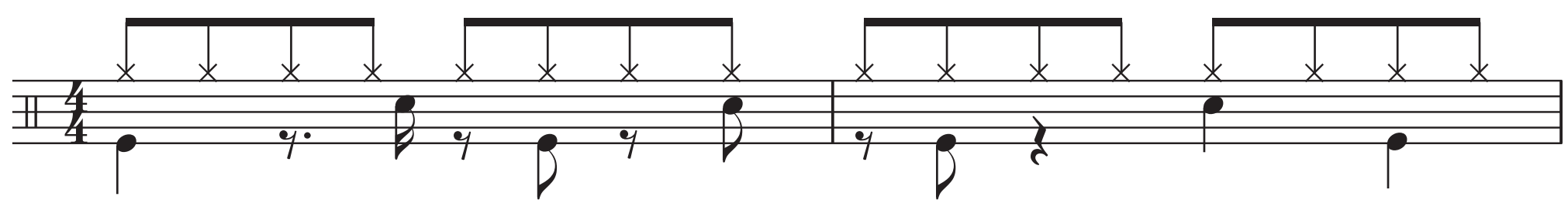
22	29	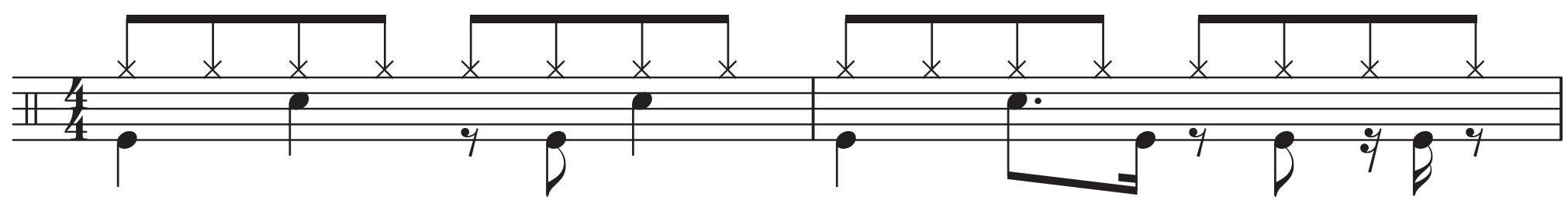
23	32	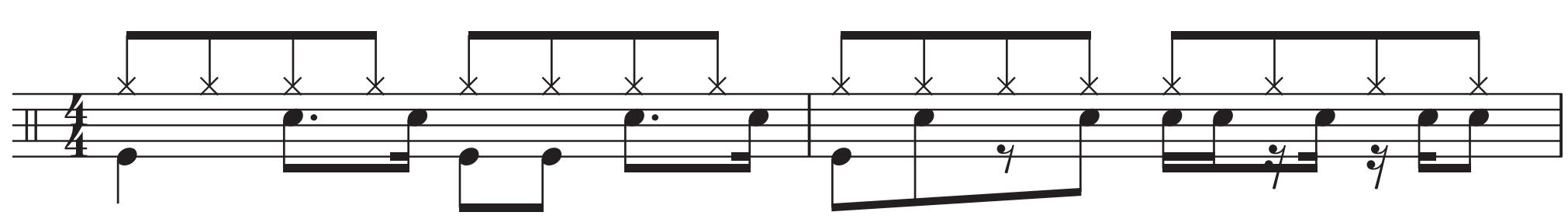
24	34	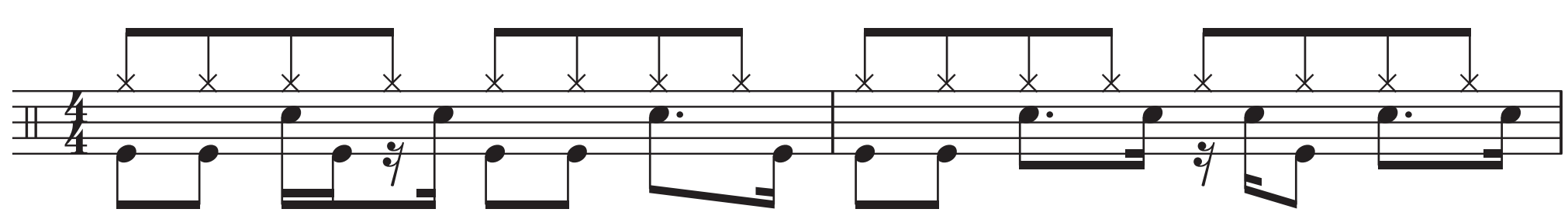

No.	SI	Rhythm sequence
25	36	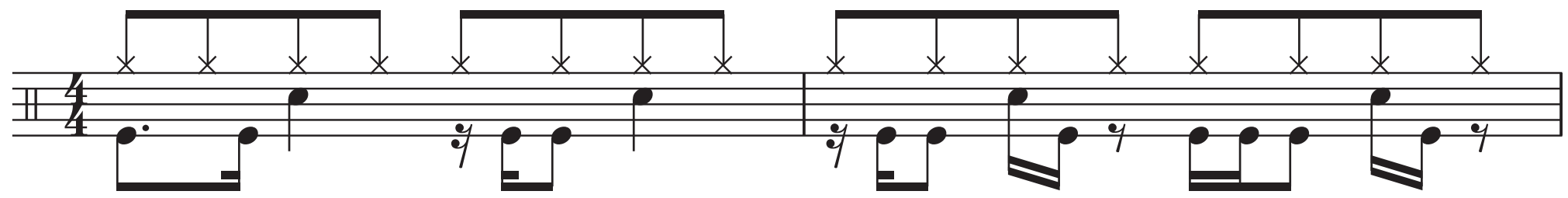
26	37	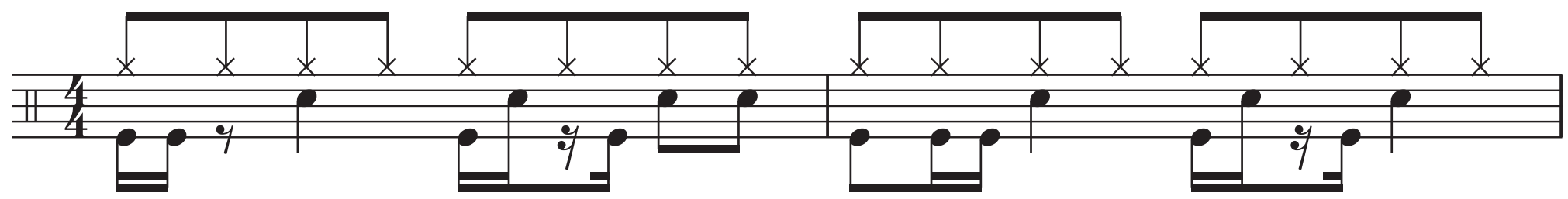
27	37	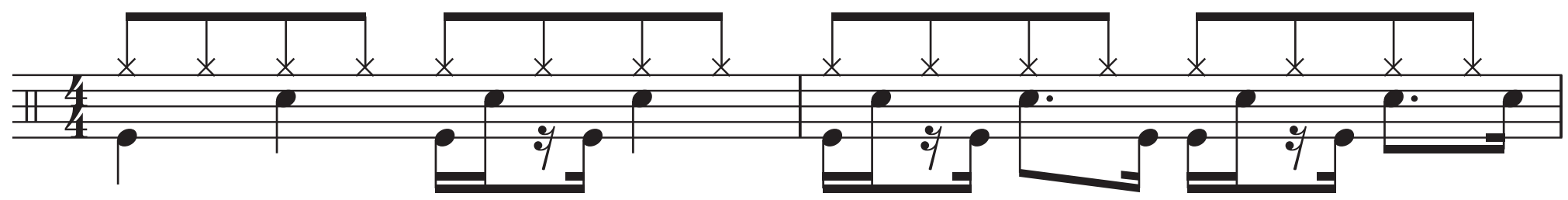
28	40	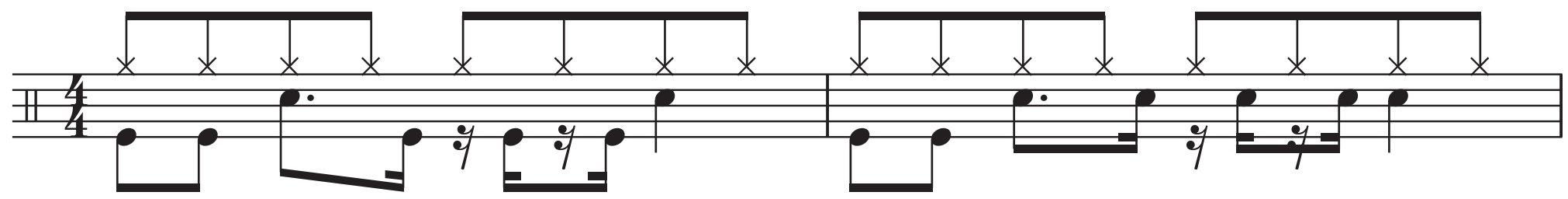
29	43	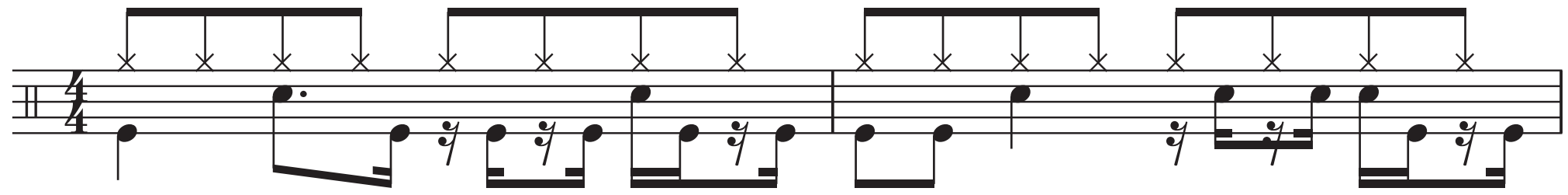
30	45	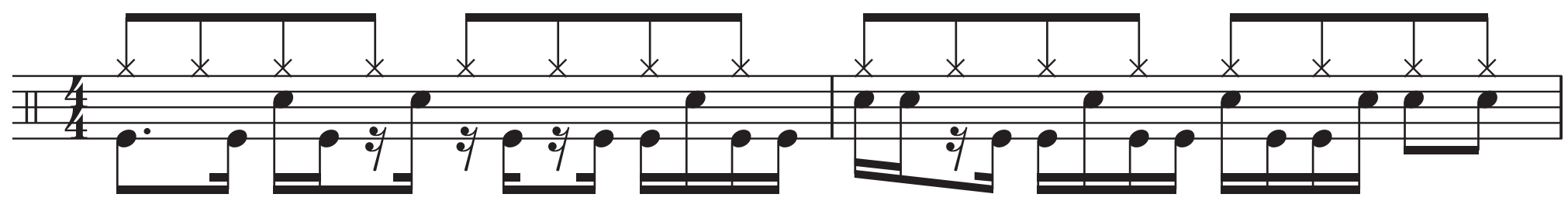
31	45	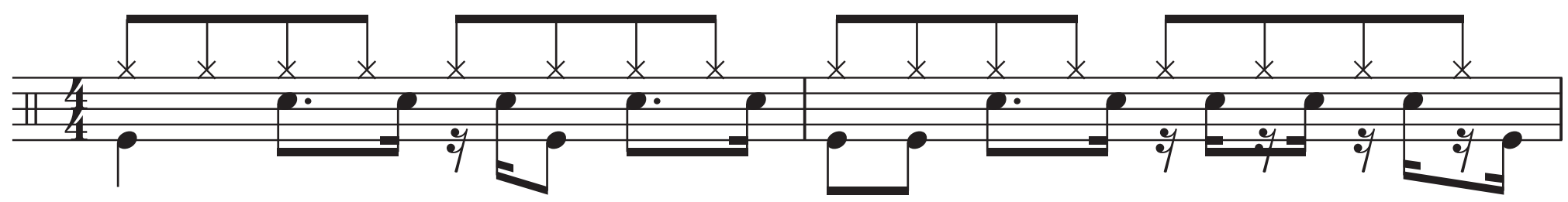
32	46	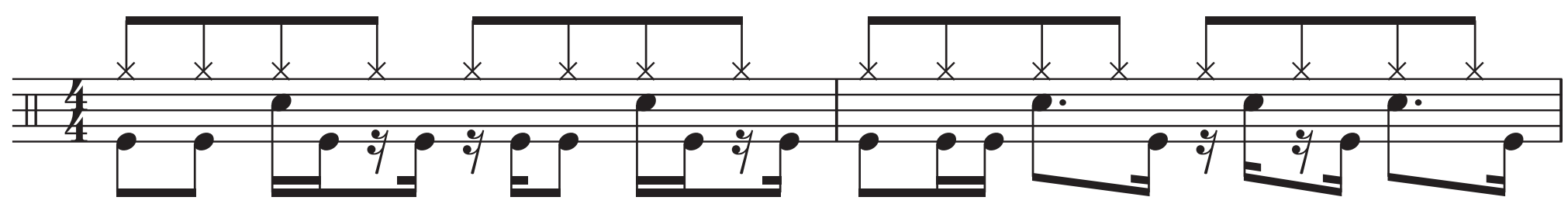
33	49	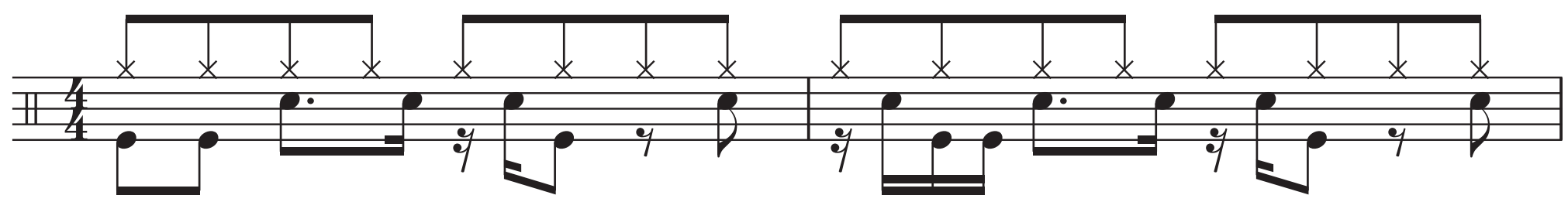
34	52	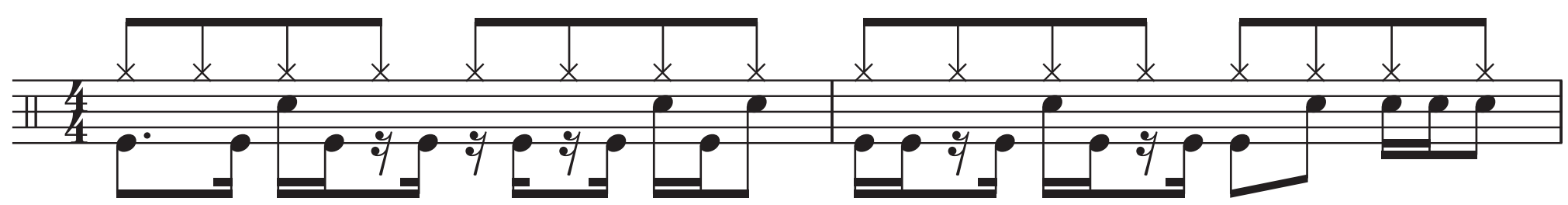
35	53	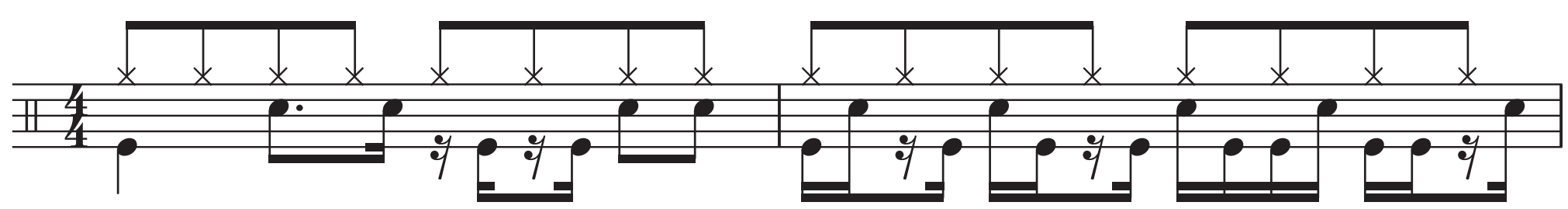
36	53	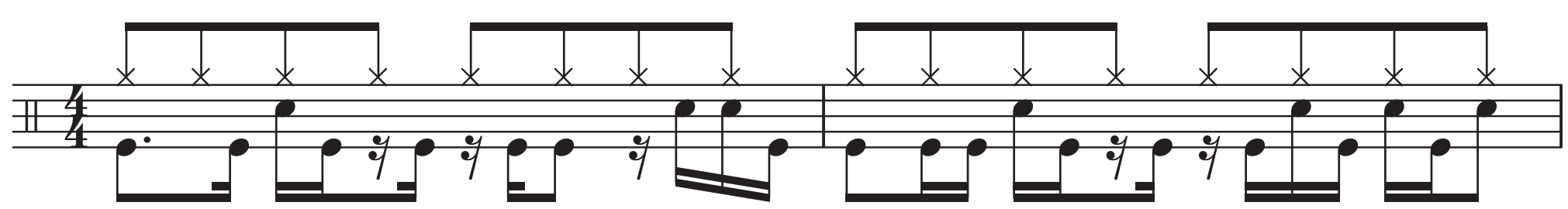

No.	SI	Rhythm sequence
37	54	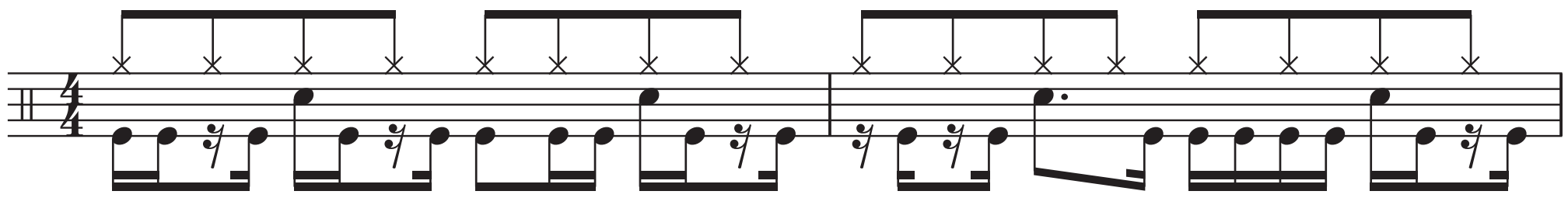
38	54	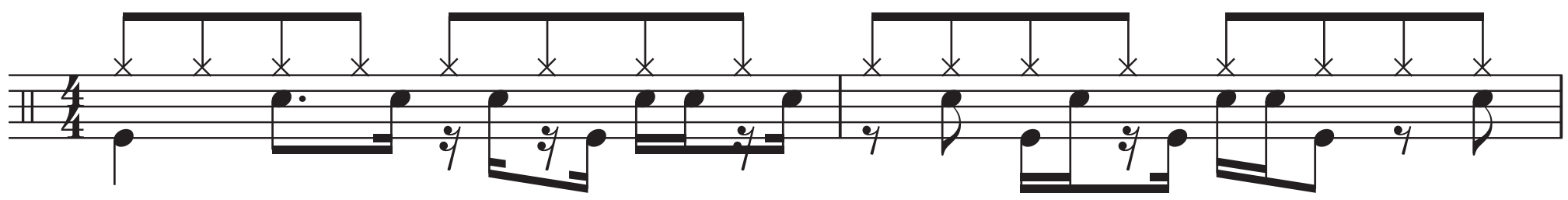
39	58	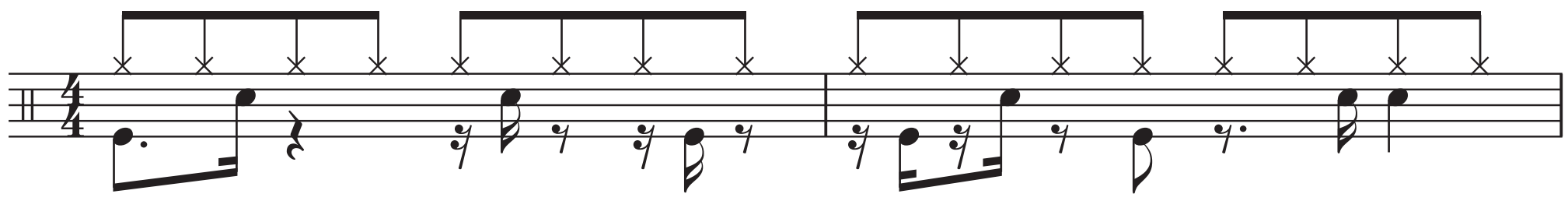
40	60	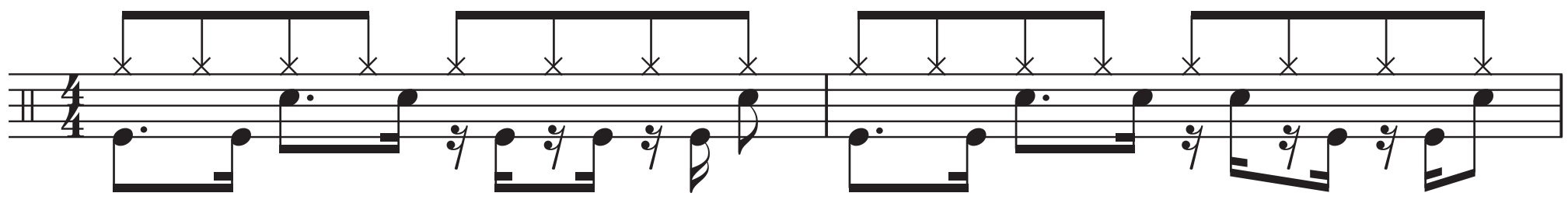
41	60	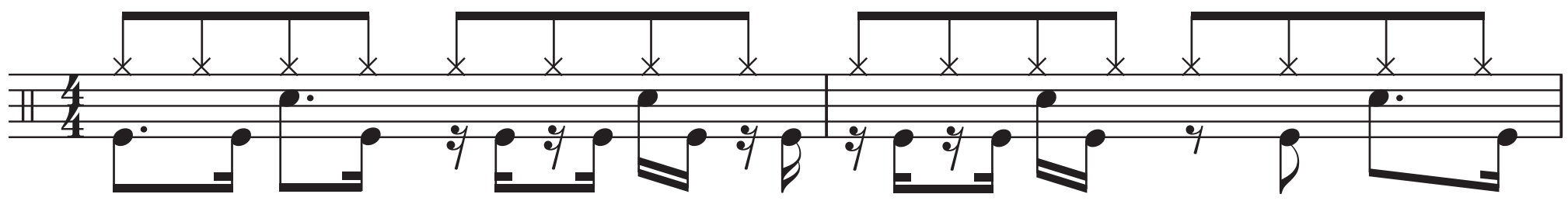
42	62	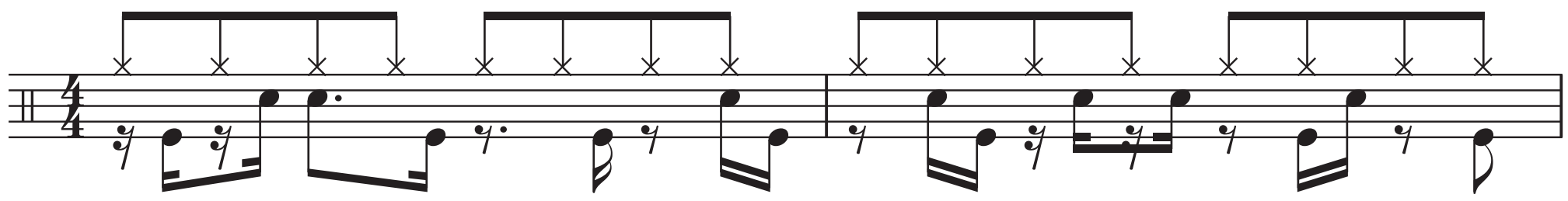
43	64	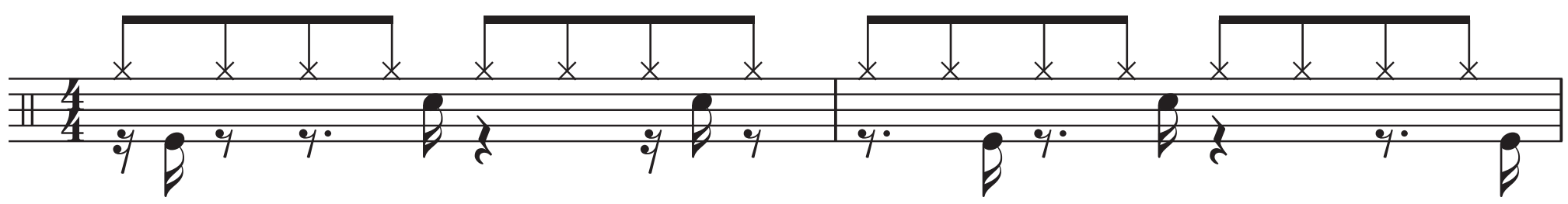
44	65	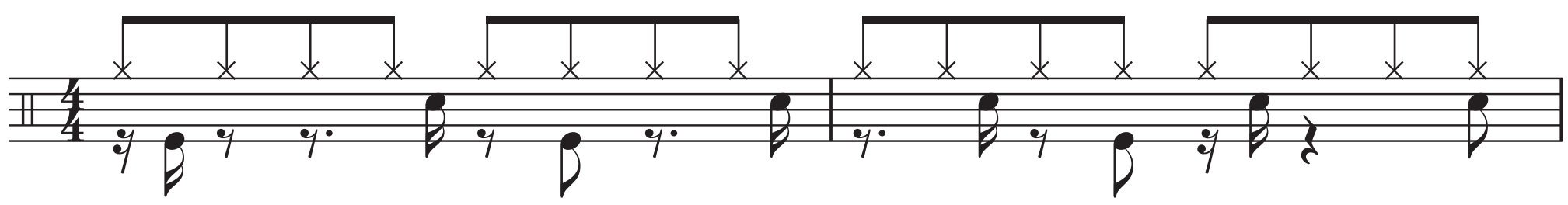
45	66	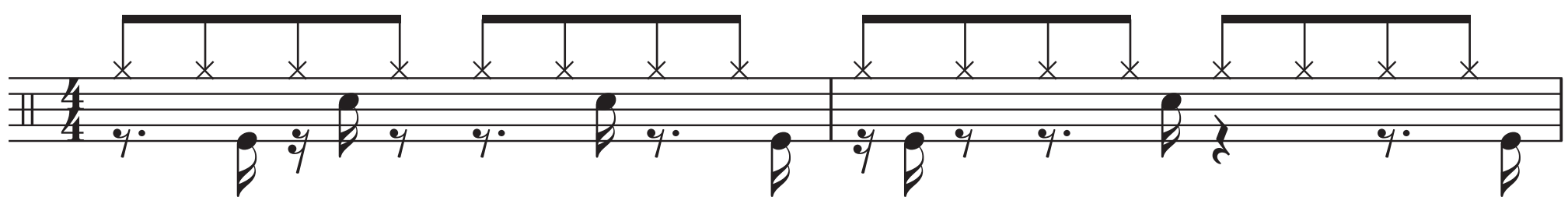
46	71	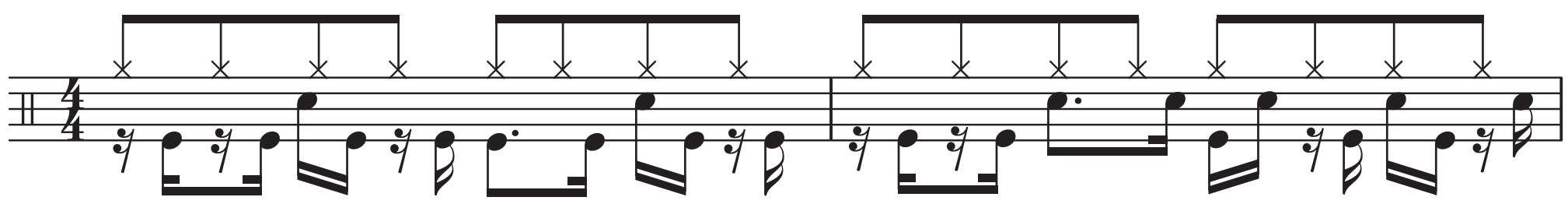
47	78	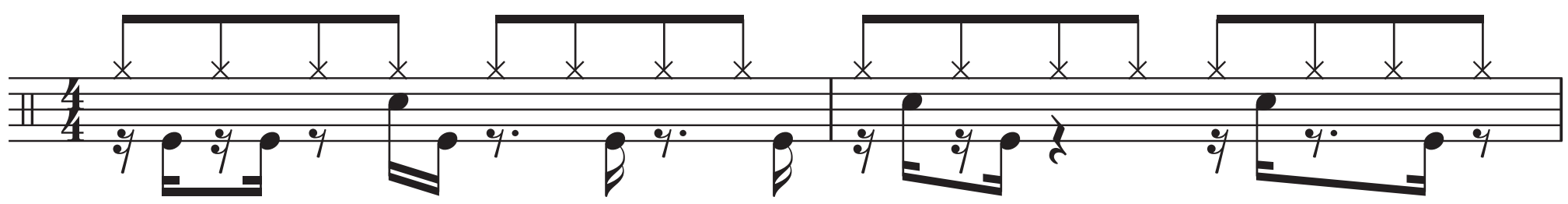
48	81	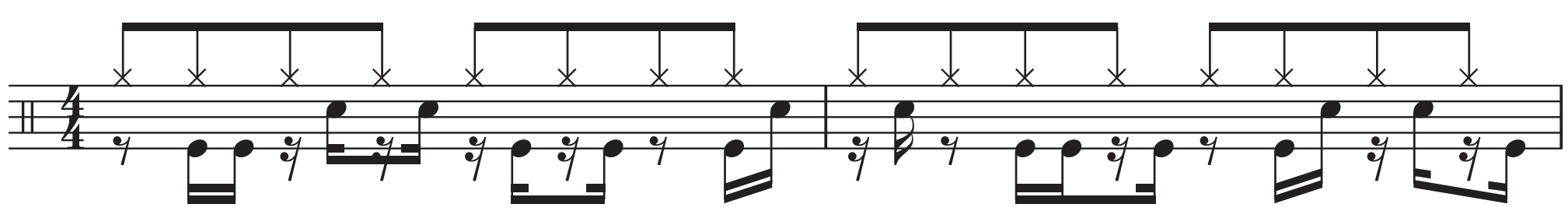
